# Prospective Study on the Correlation between CART and Leptin Gene Expression, Obesity, and Reproductive Hormones in Individuals Undergoing Bariatric Surgery

**DOI:** 10.3390/jcm13041146

**Published:** 2024-02-18

**Authors:** Charalampos Voros, Kyriakos Mpananis, Angeliki Papapanagiotou, Abraham Pouliakis, Despoina Mavrogianni, Konstantina Mavriki, Ioannis Gkaniatsos, Christina Karasmani, Ioannis Prokopakis, Menelaos Darlas, Sofia Anysiadou, George Daskalakis, Ekaterini Domali

**Affiliations:** 1 1st Department of Obstetrics and Gynecology, ‘Alexandra’ General Hospital, National and Kapodistrian University of Athens, 80 Vasilissis Sofias Avenue, 115 28 Athens, Greece; charalamposvoros@hotmail.com (C.V.); depy.mavrogianni@yahoo.com (D.M.); ntina_mrk@hotmail.com (K.M.); giannisgan@hotmail.com (I.G.); ckarasmani@gmail.com (C.K.); ioannisprokopakis@gmail.com (I.P.); mdarlas2110@gmail.com (M.D.); sofanysi@yahoo.gr (S.A.); gdaskalakis@yahoo.com (G.D.); kdomali@yahoo.fr (E.D.); 2 Ealing Hospital, London North West University Healthcare NHS Trust, 601 Uxbridge Road, Southall UB1 3HW, UK; 3 Department of Biological Chemistry, Medical School, National and Kapodistrian University of Athens, 115 28 Athens, Greece; agpana@med.uoa.gr; 4 2nd Department of Pathology, Attikon University Hospital, National and Kapodistrian University of Athens, Rimini 1, Chaidari, 124 62 Athens, Greece; apou1967@gmail.com

**Keywords:** infertility, obesity, bariatric surgery, bariatrics, CART peptide, fertility, reproductive hormones

## Abstract

Obesity, a global health concern affecting 650 million individuals of all ages worldwide, prompts health complications, including fertility issues. This research investigates the impact of bariatric surgery on morbidly obese females under 40, examining the relationship between CART and leptin gene expressions and reproductive hormones. Post-surgery, a significant reduction in BMI (16.03 kg/m^2^, n = 29) was observed, accompanied by notable hormonal changes. FSH levels showed a mean difference of 3.18 ± 1.19 pre- and post-surgery (*p* < 0.001), LH levels exhibited a mean difference of 2.62 ± 1.1 (*p* < 0.001), E2 levels demonstrated a mean difference of 18.62 ± 5.02 (*p* < 0.001), and AMH levels showed a mean difference of 3.18 ± 1.19 (*p* < 0.001). High CART and leptin expressions before treatment correlated with lower expressions after treatment. These findings, rooted in statistically significant correlations (CART: rs = 0.51, *p* = 0.005; leptin: rs = 0.75, *p* < 0.001), shed light on potential molecular pathways connecting gene expressions with reproductive hormones post-bariatric surgery. Our study uniquely investigates the interplay between genetic markers, infertility, and bariatric surgery in women. It stands out by providing distinctive insights into the development of personalized treatment strategies for obesity-related infertility, contributing to a deeper understanding of this complex medical issue.

## 1. Introduction

Obesity, defined by a body mass index (BMI) of 30 kg/m^2^ or higher, poses a complex challenge influenced by intricate interactions among genetic, socioeconomic, and cultural factors [1]. The intricate relationship involving excessive free fatty acids in the bloodstream, chronic low-grade inflammation, and metabolic disturbances underscores the complexity of obesity’s impact on normal ovarian function and fertility [2]. Bariatric surgery emerges as a viable treatment option for morbidly obese patients, especially when other approaches prove ineffective, offering efficacy in weight loss and addressing obesity-related comorbidities [3].

Establishing a panel of biomarkers, such as cocaine/amphetamine-regulated transcript (CART) and leptin, improves the comprehension of the underlying mechanisms related to appetite, energy expenditure, and obesity. Recent research suggests that obese individuals may exhibit a reduction in the CART peptide, emphasizing its role in satiety signaling and the potential consequences of its suppression on weight gain [4]. Moreover, leptin, a hormone primarily secreted by adipose tissue, plays a pivotal role in suppressing appetite and regulating energy balance by binding to specific receptors in the hypothalamus [4]. Despite the theoretical anticipation that high leptin levels in obesity should lead to reduced food intake and increased energy expenditure, the “leptin resistance” phenomenon complicates this relationship. The impaired transport of leptin across the blood–brain barrier and disrupted intracellular signaling due to chronic inflammation in obesity further underscore its intricate involvement in reproductive function [5].

The dysregulation of leptin in obesity not only affects the secretion of gonadotropin-releasing hormone (GnRH) but also influences fertility hormone production, potentially leading to infertility [6]. Moreover, leptin receptors in the ovaries directly influence ovarian follicle development [7]. Leptin activates the expression of the CART neuropeptide in the brain, indicating a significant interplay between these two regulatory factors [8]. Elevated CART levels have been observed in ovarian granulosa cells (GCs) of obese patients. Additionally, leptin stimulation increases CART expression, inhibiting GC aromatase expression and steroidogenesis [9]. Understanding variations in CART and leptin gene expression levels in response to bariatric surgery, especially sleeve gastrectomy, may reveal the mechanisms involved in appetite regulation, weight loss, and reproductive health.

Our study examines the impact of bariatric surgery on BMI decrease and aims to establish a possible link between surgical intervention, genetic markers, and hormonal alterations. CART and leptin genes may be used as biomarkers in women undergoing bariatric surgery. They may be applied for personalized therapeutic approaches, offering a valuable genetic tool for improved fertility outcomes in individuals suffering from obesity.

## 2. Material and Methods

This study includes 29 patients and has been designed and conducted strictly with ethical principles according to the Helsinki Declaration. Informed consent obtained from all participants underscores the transparency and voluntariness of their participation. The study received ethical approval from the scientific board of Alexandra General Hospital 4345, 1 March 2023 and Medical School of Athens 48859, 30 October 2020.

This prospective cohort study investigates alterations in CART and leptin expression in a specific population of obese infertile women, all aged below 40, before and six months after undergoing sleeve gastrectomy. Infertile patients are characterized by the failure to establish a clinical pregnancy after 12 months of regular and unprotected sexual intercourse. The study was conducted at Alexandra General Hospital’s gynecological tertiary care center from March 2022 until November 2023.

We included women who met certain criteria to ensure a focused and specific group. Participants had to be morbidly obese and experiencing infertility issues, with a body mass index (BMI) equal to or greater than 40 kg/m^2^. Participants had to express their intention to undergo sleeve gastrectomy as a treatment option for their morbid obesity. This approach ensured that our study included women with relevant characteristics to our research objectives.

**Figure 1:**
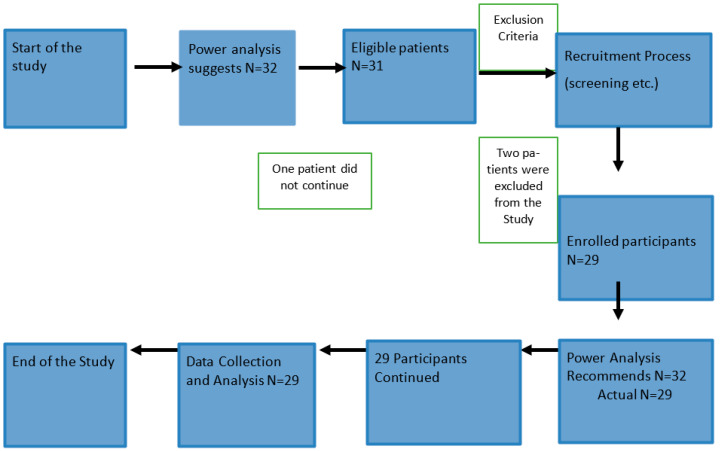
Flow chart of patients’ elimination and inclusion in the study.

To minimize potential confounding factors, we excluded certain groups from the study (Figure 1). Women above 40 were excluded to maintain consistency within the reproductive age group. This decision ensured that our results would apply to patients in their reproductive years. Participants with significant medical conditions impacting fertility or gene expression were excluded to maintain a more homogeneous participant population. Furthermore, to decrease interference with our outcomes of interest, the study did not include women taking medications known to affect fertility or gene expression, such as certain hormonal therapies or immunosuppressants. After surgery, patients typically stayed in the hospital for 1–2 days. Postoperatively, they started a liquid diet and gradually transitioned to solid foods over several weeks.

The study involved the collection of blood samples from the participants both before and six months after sleeve gastrectomy. These samples were analyzed to evaluate the expression levels of CART and leptin, as well as other applicable reproductive hormones, including follicle-stimulating hormone (FSH), luteinizing hormone (LH), estradiol (E2), sex hormone-binding globulin (SHBG), anti-Müllerian hormone (AMH), and free testosterone. Blood samples were collected in the morning after an overnight fast to ensure consistent hormonal levels.

## 3. Detection of Leptin and CART Gene Expression

Blood samples were collected from all women before surgery and 6 months later and were immediately stored at −80 °C until RNA extraction. Total RNA was extracted from blood samples using the Monarch Total RNA miniprep kit provided by New England Biolabs (Ipswich, MA, USA). Complementary DNA (cDNA) was then synthesized using 1 μg from the extracted RNA using the LunaScript RT SuperMix, also from New England Biolabs. To assess the gene expression of leptin and CART, we utilised real-time polymerase chain reaction (RT-PCR) using 5 μL of cDNA. All RT-PCR reactions were conducted on a Light-Cycler480II instrument manufactured by Roche Life Sciences, utilizing the Luna Universal qPCR Master Mix from New England Biolabs at 1× final concentration. All sequence-specific primers were purchased from Eurofins Genomics GmBH (Germany). The sequences of the CART gene primers were forward primer: 5′GCTGAAGAAGCTTTGAAGAAGC3′ and reverse primer: 3′GCACTTCAGGAGGAAGGAATTGC5′. For the leptin gene, the primers were forward primer: 5′GAACCCTGTGATTCTT3′ and reverse primer: 5′CCAGGTCGTTATTTGG3′.

The PCR reaction conditions consisted of an initial denaturation step at 94 °C for 1 min, followed by 40 cycles of denaturation at 95 °C for 15 s and annealing/extension at 60 °C for 30 s. Subsequently, a melting curve analysis was performed to validate the specificity of the results. To further confirm the specificity of the PCR reaction, we also verified the size of the amplified product using conventional agarose gel electrophoresis. The G6PD gene was also used as a housekeeping gene for normalization purposes. Each assay was run in duplicate to ensure the accuracy and reliability of the results, and a negative control was included to account for any potential contamination or background signal. The 2^−ΔΔCT^ method was used to calculate the relative mRNA expression levels of the CART and leptin genes.

## 4. Sample Size Determination

A power analysis was conducted to estimate the appropriate sample size for our study and to ensure that the study had sufficient statistical power to detect meaningful differences in these outcomes. We thoroughly reviewed the existing literature and consulted relevant studies to evaluate the power analysis’s effect size. Based on this literature review and expert consultation, we assumed a moderate effect size (Cohen’s d = 0.5) for the primary outcome variables, CART and leptin gene expression. This effect size was considered a conservative estimate, allowing the detection of meaningful changes in gene expression. A desired statistical power level of 80% (1 − β = 0.80) was selected to provide a high likelihood of detecting true effects if they existed. The significance level (α) was set at 0.05, representing a 5% risk of a type I error. We planned to use paired *t*-tests to compare the means of CART and leptin gene expression levels before and six months after bariatric surgery, non-parametric tests for paired data, and the Wilcoxon signed-rank test. We also considered the association between the pre- and post-surgery measurements, assuming a moderate correlation coefficient of 0.5 based on similar studies. Using these parameters, we used statistical software (i.e., G*Power, version 3.1.9.7) to conduct the power analysis. According to that analysis, we revealed that a total sample size of 29 participants would be necessary to have the desired statistical power (32 when assuming a 10% loss of follow-up). Practical considerations, such as patient availability and resources, were taken into account to ensure the feasibility of the study within the clinical setting.

## 5. (Methods) Statistical Analysis

Data were recorded in Microsoft Excel (version 2401, 2016) spreadsheets (Microsoft Corporation, Redmond, Washington, DC, USA) in rows corresponding to each patient. Statistical analysis was performed via the SAS for Windows 9.4 software platform (SAS Institute Inc., Cary, NC, USA). Descriptive data were expressed as mean value and standard deviation (SD). We calculated the differences in the levels of hormones as their value after surgery minus the value before surgery (therefore, a positive value is linked with increment and a negative with reduction); in the statistics arena, we applied non-parametric tests for paired data, specifically the Wilcoxon signed-rank test. The numbers of patients who experienced an increment or reduction in the hormonal values and the relevant percentages were also calculated. To investigate whether the reduction (or increment) in hormones was linked with a reduction or increment of the BMI as well as to the studied gene expressions, we calculated the Spearman correlation coefficient (rs) (a positive value near to 1 indicates a strong positive correlation while a negative value nearer to −1 a strong negative role). The significance level for the study was set to *p* < 0.05, and all tests were two-sided.

## 6. Results

### 6.1. Changes in Hormones

The study involved 29 women following sleeve gastrectomy because of morbid obesity, who were monitored 6 months post-surgery. Their clinical and biochemical characteristics are shown in Table 1.

Twenty-nine patients participated in the study; their mean age was 33.0 years (SD: 4.1 years, range: 26–39 years). Their mean weight and height were 109.89 kg (SD:19.28) and 1.53 m (SD: 0.21), respectively. Their BMI was 41.94 kg/m^2^ (SD: 3.98).

Table 2 presents the mean and standard deviation of the hormone levels before and after surgery and their differences. The mean reduction in BMI was 16.03 kg/m^2^, and all patients experienced similar BMI reduction. Moreover, all patients experienced an increase in hormone levels except free testosterone, which in all cases was reduced. Table 2 offers a detailed comparison of various medical measurements before and after sleeve gastrectomy. Our primary findings detail reproductive hormones FSH, LH, E2, SHBG, and AMH levels. All experienced a significant improvement post-sleeve gastrectomy.

Specifically, FSH levels rose from an average of 5.99 mIU/mL to 9.16 mIU/mL, LH from 6.35 to 8.97 mIU/mL, E2 from 29.81 to 48.43 pg/mL, SHBG from 36.24 to 64.19 nmol/L, and AMH from 2.14 to 2.96 ng/mL. On the other hand, free testosterone, BMI, and Cpt0 CART levels decreased remarkably. The free testosterone level dropped from 28.72 ng/dL to 9.42 ng/dL, BMI decreased from a pre-surgery value of 41.94 to 25.91, and the adjusted Cpt0 CART value went from 0.3 to −3.42. As for Cpt0, leptin displayed a mixed response. Its level increased overall from −1.81 to −0.13 when adjusted; the percentage change reveals an 89.66% increase and a 10.34% decrease. According to all these results, the consistent *p*-value of <0.0001 implies that the observed changes are statistically significant and not coincidences.

### 6.2. BMI Correlation to Hormone and Gene Expressions

This section examines correlations of hormone levels to BMI before and after the intervention. Considering BMI, presurgical BMI did not correlate with the relevant BMI after surgery (rs = 0.089, *p* = 0.650), although, as mentioned in the previous section, it was decreased for all patients. In contrast, certain important correlations were observed between some hormones (specifically FSH, LH, and E2) and both genes’ expressions before the intervention, and BMI after the intervention was found to correlate with E2 and again both gene expressions. Specifically, BMI before surgery was negatively related to FSH (rs = −0.58, *p* = 0.001), LH (rs = −0.41, *p* = 0.028), and E2 (rs = −0.38, *p* = 0.046), while post-surgical BMI was found negatively related with E2 (rs = −0.42, *p* = 0.025) and we expect that with a larger sample additional correlations would be obtained with FSH and LH (rs = −0.30, *p* = 0.116 and rs = −0.27, *p* = 0.171), respectively. A negative correlation (rs = −0.417, *p* = 0.027) existed between BMI and the expression of the CART gene before intervention. This suggests that as BMI increases, the expression of this gene tends to decrease.

On the contrary, there was a positive correlation (rs = 0.458, *p* = 0.014) for leptin expression, meaning that as BMI increases, the expression of this gene increases. After the sleeve gastrectomy, as the BMI tends to decrease, there is a pronounced increase in the expression of the CART gene as indicated by the positive correlation (rs = 0.552, *p* = 0.002), and the expression of the leptin gene shows a decrease as indicated by the negative correlation (rs= −0.541, *p* = 0.002). 

Table 3 presents the correlation coefficient of the adjusted CART and leptin expression before surgery, with the hormone levels measured simultaneously. Significant correlations were reported for the expression of CART with FSH, LH, and E2 (rs = −0.58, −0.45, and −0.68 with *p* < 0.05 in all cases). Similarly, the post-surgical results are presented in Table 4; strong FSH, LH, and E2 correlations were found with CART and leptin.

### 6.3. Relations between the Two Gene Expressions

When CART gene expression was detected before and after bariatric surgery, a positive fold change was detected in 22 out of 29 samples, corresponding to up-regulation of the gene. A maximum up-regulation of 2.49 was revealed, while 7 of 29 showed negative expression related to down-regulation of the gene. When leptin gene expression was detected before and after bariatric surgery, a positive fold change was detected in 25 out of 29 samples, corresponding to up-regulation of the gene. A maximum up-regulation of 2.5 was revealed, while 2 of 29 showed negative expression related to down-regulation of the gene. A comparison of the expression of both genes demonstrated a positive correlation between them before and after bariatric surgery. Up-regulation of leptin was associated with up-regulation of the CART gene, revealing an induction of gene expression.

Finally, we investigated all possible correlations between the two gene expressions before and after the intervention (Table 4). A high CART expression before treatment was linked to a statistically significant difference in lower CART expression after treatment (rs = 0.51, *p* = 0.005, see Table 4); this was also found for leptin (rs = 0.75, *p* < 0.001). Finally, CART and leptin expressions after surgery were also highly correlated (rs = 0.67, *p* < 0.001), while this was not found before treatment (rs = 0.17, *p* = 0.383).

## 7. Discussion

Treatment options for obesity encompass various approaches, which can be broadly classified into non-surgical and surgical methods. Bariatric surgery stands out as a highly effective option with promising outcomes, including prevention of diabetes mellitus and decreased risk of cardiovascular diseases. Additionally, it seems that it improves the hormonal status of the patients [10,11,12]. In our study, the individuals underwent bariatric surgery to reduce body weight; more specifically, we performed a sleeve gastrectomy. This operation is characterized by the resection and removal of most of the gastric body, leading to the alteration of the hormonal profile [13]. The operation was chosen for the patients because of the advantages it offers. The simplicity of the procedure is one of the benefits compared with other forms of bariatric surgery such as the gastric bypass [14]. Moreover, no foreign objects (like a band or balloon) are left inside the body. Along with the stomach’s reduced size, some patients decrease hunger hormone (ghrelin) production, potentially reducing appetite [15].

Our study provides information on changes in reproductive hormones and associated gene expression domains following bariatric surgery. Patients experienced statistically significant FSH, LH, and E2 levels 6 months after the intervention. Our research did not observe statistically significant improvements in free testosterone, SHBG, or AMH levels. Sarwer DB. et al. reported statistically significant differences in the expression of FSH and LH but not in E2 levels in their prospective cohort study of 106 morbidly obese women who underwent bariatric surgery [16]. CART expression is important in morbid obesity and the hormonal pathways in animal models. Asnicar et al. examined CART-deficient mice and observed that when they were fed a high-fat diet, they exhibited an increased tendency to become obese [13]. Lee et al. showed in their study that CART injection into the nucleus tractus solitarii reduces food intake in obese rats [17]. Kristensen et al. demonstrated that CART peptides acted against feeding, even when NPY (neuropeptide Y), a prominent stimulator of appetite in the central nervous system, was administered [8]. Okumura et al. and Asakawa et al. both observed that injection of CART into the fourth ventricle led to a reduction in the rate of gastric emptying, similar to the effects seen with corticotropin-releasing factor (CRF) and cholecystokinin [18,19]. In their study, Patkar et al. investigated the association between bariatric surgery and CART expression in mice with morbid obesity. The mice were divided into four subgroups depending on the procedure. The first group underwent RYGB surgery, the second sleeve surgery, the third caloric restriction, and the last control group were not operated on. The findings did not show any statistically significant differences in mRNA expression of CART peptide among the four groups.

Furthermore, the study proposed that RYGB affects hypothalamic genes differently, from caloric restriction to weight loss [20]. In their studies, Grayson et al. and Cavin et al. reported no statistically significant differences in CART expression after bariatric surgery in morbid obese rats [15,21]. Patkar et al. showed that CART levels remained unchanged before and after bariatric surgery, with no significant differences in mRNA expression between the chow-fed and RYGB groups [20]. Furthermore, Muñoz-Rodríguez et al. observed that before bariatric surgery, obese individuals exhibited higher CART levels compared with normoweight. However, there was no statistically significant difference one year after surgery [22]. Singh et al. 2021, in their review, suggest that CART peptide is a potential therapeutic option for morbidly obese individuals [23].

The role of leptin in the ovary in relation to obesity-related infertility has been a matter of ongoing debate in recent years [24,25]. Both in vitro and in vivo studies have shown that leptin negatively affects ovarian steroidogenesis. It modulates the production of estrogens and progesterone and is important in regulating follicle development [26]. Anifantis et al. showed that patients who underwent IVF with low serum and follicular fluid leptin levels were associated with more “good quality” embryos, enhanced implantation, and higher pregnancy rates [6]. Xiaoting Ma et al., in their investigation, proposed that heightened leptin levels in obesity trigger an increase in CART within ovarian follicle granulosa cells. Additionally, this increase disrupts intracellular cAMP levels, MAPK signalling, and aromatase gene expression. As a result, there is a decrease in estradiol production and a reduction in the number of ovulated oocytes [9].

To the best of our knowledge, our study is the first to detect the mRNA expression of CART and leptin in infertile obese women before undergoing bariatric surgery and six months later. In our study, we observed that the CART gene was expressed in all samples before surgery, with 22 out of 29 samples showing significant gene expression. Similarly, leptin was expressed in 25 out of 29 samples. The expression of both genes was found to be statistically significantly different before and 6 months after the procedure. Before the operation, the expression patterns of both genes were similar. After the operation, expression of the leptin gene was observed at higher levels than the CART gene. These findings provide valuable insights into the potential impact of bariatric surgery on the expression of CART and leptin genes in obese infertile patients. Our results may be applied in the establishment of a genetic profile related to women with obesity and infertility problems. Apart from being a less invasive method for detecting gene expression, this may also lead to a more precise and personalized medical approach.

Our study included women with a BMI > 40 kg/m^2^ [27]. Although we collected blood samples before and after the bariatric surgery, we could not repeat the experiment one year later to investigate the gene expression further. We also did not detect the circulating levels of leptin and CART proteins or extract RNA from adipose tissue. Both these experimental approaches should provide us with extended information on the protein levels and gene expression in adipose tissue. A future proposal could be to compare and correlate the expression of leptin and CART genes in blood and adipose tissue before and after bariatric surgery. It would also be of great interest to investigate the methylation patterns of both genes before and after surgical intervention, as it seems that the increase or decrease in their expression depends highly on the methylation mechanisms. CART protein has been reported to act against reactive oxygen species (ROS) [28]. ROS may damage proteins, lipids, and nucleic acids, affecting fertility mechanisms. Thus, CART’s presence in mitochondria may have protective effects [29]. Consequently, a future proposal may be the study of oxidative stress mechanisms in women before and after bariatric surgery and their relation with the expression of the CART gene [29]. Little is known about the presence of single nucleotide polymorphisms in CART and leptin genes. Studying possible interactions between SNPs and gene expression would be of great interest. CART gene expression will help us transform clinical and personalized science [30].

## 8. Conclusions

Obesity, a corrosive health challenge recognized globally, continues its unsettling ascension in prevalence. Bariatric surgery, sleeve gastrectomy, is being hailed as a pivotal process in dealing with morbid obesity. Its reputed benefits span not only substantial weight reduction but also potential enhancements in fertility. This puzzle nudges us to investigate the sophisticated association of neural and endocrine pathways inherent to obesity.

The neuropeptide CART (cocaine and amphetamine-regulated peptide) has emerged as a potential key player. Its expression appears modulated by shifts in body mass index (BMI). Against this backdrop, our study underlines the imperative of investigating the ripple impacts of BMI adjustments on CART gene expression in morbid obese female patients after sleeve gastrectomy. We investigated the expression of both the CART and leptin genes from the pre-operative phase to a six-month post-operative juncture. Our investigation pronounced a statistically significant alteration in reproductive hormones post-sleeve gastrectomy. The post-operative hormonal tableau showcased transformative shifts, potentially heralding a profound rearranging of hormone levels after the intervention.

Reproductive hormones such as estrogen, luteinizing hormone (LH), and follicle-stimulating hormone (FSH) are cornerstones of fertility. Alterations in their equilibrium can have a cascade effect on ovulation, menstrual regularity, and broader reproductive health. Central to our exploration was the nexus between these hormonal oscillations and the expressions of the CART and leptin genes—a statistically significant association between the hormones and the CART and leptin genes post-sleeve gastrectomy. The previous literature has hinted at the roles of leptin and CART in reproductive endocrinology. The correlation between the alterations in CART and leptin expressions and reproductive hormone levels post-surgery opens up a rich tapestry of research avenues. The intriguing possibility arises: are these gene expressions acting as mediators or regulators in the observed post-surgery hormonal profile?

Further complicating this complex narrative, while CART’s relationship with appetite and obesity has been substantially explored in preclinical research, there remains a significant void in the clinical literature addressing its expression shifts post-sleeve surgery in humans. Our study conducted an inquiry into understanding how sleeve-induced adipose tissue reductions influence these gene expressions. This research could herald a paradigm shift in obesity and reproductive health management. The present study proposes that individual variations in CART and leptin expressions may influence responses to IVF treatment. Our results underline the positive impact of bariatric surgery on BMI reduction and the potential link between surgical intervention, genetic markers, and hormonal changes and consequently with infertility issues. These significant correlations may be applied to personalized therapeutic approaches, offering a valuable genetic tool for improved fertility outcomes in obese individuals.

## Figures and Tables

**Table 1:** Clinical characteristics of the women included in the study.

Characteristics	Mean ± SD
Age (years)	33.0 ± 4.1 (26, 39)
Weight (kg)	109.89 ± 19.28
Height (m)	1.53 ± 0.21
BMI (kg/m^2^)	41.94 ± 3.98
LH (mIU/mL)	6.35 ± 0.67
E2 (pg/mL)	29.81 ± 2.65
SHBG (nmol/L)	36.24 ± 7.58
Free testosterone (ng/dL)	28.72 ± 9.35
AMH (ng/mL)	2.14 ± 0.24
Duration of infertility (years)	3 (2, 5)
Cpt0 CART (adjusted)	0.3 ± 4.41
Cpt0 Leptin (adjusted)	−1.81 ± 1.82

**Table 2:** Mean values and standard deviation of hormone levels and gene expression before and after surgery and the mean values of differences. Column percentage is related to the number of patients that had increased, decreased, or stable levels (indicated by ↑, ↓ and—respectively).

	Before	After	Deference (After–Before)		*p*-Value
Measure	Mean ± SD	Mean ± SD	Mean ± SD	Percentage	
FSH (mIU/mL)	5.99 ± 1.11	9.16 ± 0.72	3.18 ± 1.19	↑ 100%	<0.0001
LH (mIU/mL)	6.35 ± 0.67	8.97 ± 0.94	2.62 ± 1.1	↑ 100%	<0.0001
E2 (pg/mL)	29.81 ± 2.65	48.43 ± 5.41	18.62 ± 5.02	↑ 100%	<0.0001
SHBG (nmol/L)	36.24 ± 7.58	64.19 ± 12.41	27.95 ± 8.87	↑ 100%	<0.0001
Free testosterone (ng/dL)	28.72 ± 9.35	9.42 ± 2.52	−19.3 ± 9.15	↓ 100%	<0.0001
AMH (ng/mL)	2.14 ± 0.24	2.96 ± 0.15	0.82 ± 0.19	↑ 100%	<0.0001
BMI (before surgery)	41.94 ± 3.98	25.91 ± 1.5	−16.03 ± 4.24	↓ 100%	<0.0001
Cpt0 CART (adjusted)	0.3 ± 4.41	−3.42 ± 1.14	−3.72 ± 4.3	↓ 100%	<0.0001
Cpt0 Leptin (adjusted)	−1.81 ± 1.82	−0.13 ± 1.55	1.68 ± 1.2	↑ 89.66%, ↓ 10.3%	<0.0001

**Table 3:** Correlation of CART and leptin with hormone levels, pre- and post-surgery.

	Before Surgery				After Surgery			
	CART		Leptin		CART		Leptin	
	rs	*p*	rs	*p*	rs	*p*	rs	*p*
FSH (mIU/mL)	−0.577	**<0.001**	0.161	0.412	−0.619	**<0.001**	−0.758	**<0.001**
LH (mIU/mL)	−0.450	**0.016**	−0.120	0.542	−0.600	**<0.001**	−0.534	**0.003**
E2 (pg/mL)	−0.680	**<0.001**	−0.080	0.679	−0.656	**<0.001**	−0.651	**<0.001**
SHBG (nmol/L)	−0.048	0.807	0.085	0.666	0.181	0.356	0.128	0.515
Free testosterone (ng/dL)	−0.092	0.64	−0.023	0.903	−0.278	0.151	0.032	0.868
AMH (ng/mL)	0.108	0.583	−0.078	0.691	0.053	0.785	0.101	0.608

**Table 4:** Correlation coefficients of CART and leptin between them as well as before and after the intervention. In each cell, the first number is the Spearman correlation coefficient and the second number is the *p*-value (in bold when significant).

	CART (before)	CART (after)	Leptin (before)	Leptin (after)
CART (before)		0.515, *p* = **0.005**	0.171, *p* = 0.383	0.224, *p* = 0.249
CART (after)	0.515, *p* = **0.005**		0.603, *p* **< 0.001**	0.668, *p* = **<0.001**
Leptin (before)	0.171, *p* = 0.383	0.603, *p* **< 0.001**		0.752, *p* **< 0.001**
Leptin (after)	0.224, *p* = 0.249	0.668, *p* **< 0.001**	0.752, *p* = **<0.001**	
